# Unraveling the cardiac consequences of hypothyroidism: a case report of sinus arrest and bradycardia exacerbated by seasonal changes, escitalopram and medication noncompliance

**DOI:** 10.1093/omcr/omaf247

**Published:** 2025-11-26

**Authors:** May Thu Kyaw, James Smitt

**Affiliations:** Heart and Vascular Center, Victoria hospital, Mayangone township, 11061 Yangon, Myanmar; Internal Medicine, University of Medicine 2, Yangon, Myanmar; Internal Medicine, University of Medicine 2, Yangon, Myanmar; Internal Medicine, OSC Hospital, Yangon, Myanmar

**Keywords:** hypothyroidism, bradycardia, sinus arrest, thyroxine, pacemaker

## Abstract

Thyroid hormone is essential in human metabolism. Hypothyroidism causes bradycardia, sinus pause, sinus arrest, and the most severe form, myxedema coma, and cardiopulmonary arrest. Most cardiovascular manifestations secondary to hypothyroidism are reversible with timely thyroid hormone replacement. Herein, we report a case of a 53-year-old woman with hypothyroidism who experienced episodes of prolonged sinus arrest, which resolved following temporary pacing and initiation of levothyroxine therapy. Factors such as seasonal variation, medication noncompliance, recent viral illness, and concurrent escitalopram use likely contributed to her presentation. This case highlights the importance of prompt recognition and management of hypothyroid-related cardiac arrhythmias.

## Introduction

Worldwide prevalence of hypothyroidism ranges from 4%–10%. However, it may be underestimated because most cases are subclinical [1]. Clinical features of hypothyroidism are chronic and non-specific. It can present with various cardiovascular abnormalities like conduction problems, pericardial effusion, and diastolic hypertension. However, sub-clinical or inadequately treated hypothyroidism can acutely present with overt life-threatening complications such as myxedema coma and cardiac arrest when precipitated by various factors such as infection, cold weather, medications (sedatives, narcotics, some antidepressants), and surgery [2, 3].

## Case presentation

A 53-year-old lady presented to the emergency department with a history of syncope in the early morning of June. Physical examination showed a slightly obese female (BMI 30 kg/m^2^) with facial puffiness, dry coarse skin, and non-pitting edema. Her vital signs included blood pressure 130/90 mmHg, heart rate 45 bpm, SpO₂ 99% on room air, respiratory rate 18 breaths/min, and temperature 36.5°C. The neurological examination was unremarkable, with a GCS of 15/15. Cardiorespiratory examination was unremarkable.

### History

She has a history of hyperthyroidism 15 years ago, treated with Carbimazole for 2 years followed by a single dose of Radioiodine. Over the last 3 years, she had been diagnosed with hypothyroidism, with elevated thyroid-stimulating-hormone (TSH) and low free triiodothyronine (T3). Since then, she has been on oral Thyroxine supplements daily but with poor compliance. Moreover, She had been on oral Escitalopram 10 mg daily for generalized anxiety disorder for the past two years, without regular thyroid function monitoring. Recently, she experienced flu-like symptoms for two days prior to admission. She also suffered from effort intolerance, fatigue, and constipation.

### Investigations

**Figure 1:**
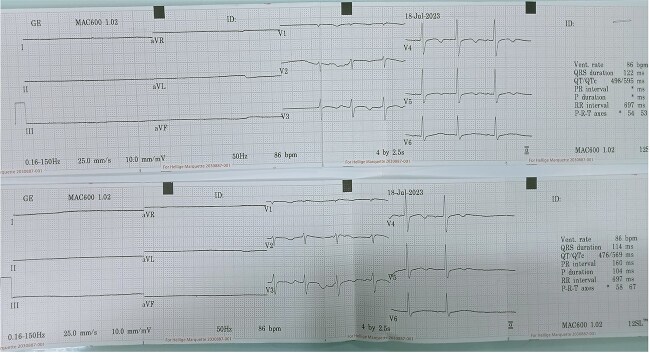
ECG showing heart rate 85/min with sinus arrest.

Initial electrocardiogram (ECG) showed a heart rate of 86/min with sinus arrest (Fig. 1). She is not taking any atrioventricular (AV) nodal-blocking agents such as beta-blockers, calcium channel blockers, or digoxin.

Laboratory results showed elevated TSH (20.15 μIU/ml) and low free T3 (1.2 pmol/L), confirming hypothyroidism. Serum calcium level was normal (Table 1). Echocardiography showed left ventricular ejection fraction (LVEF) 55% with increased pericardial fat without pericardial effusion.

**Table 1:** Laboratory test results.

Name of Test	Patient’s Value	Reference Range
Total white cell counts	13.40	4.00–11.00 x 10^9^/L
Hemoglobin	10.8	11.0–16.5 g/dl
Platelet	278	150–400 x 10^9^/L
Urea	6.4	2.7–8.0 mmol/l
Sodium	138	135–145 mmol/l
Potassium	4.23	3.60–5.20 mmol/l
Chloride	102.8	98.0–107.0 mmol/l
Bicarbonate	23.2	22.0–29.0 mmol/l
Creatinine	76	44–84 micromole/L
eGFR	69.0	≥60 mL/minute/1.73 m^2^
Free T3	1.2 ↓	3.10–6.80 pmol/L
Thyroid stimulating hormone (TSH)	20.15 ↑	0.270–4.200 μIU/ml
Corrected Calcium	2.34	2.15–2.65 mmol/l
Total Cholesterol	234.2 ↑	≤200 mg/dl
Triglyceride	140.0	≤150 mg/dl
HDL-Cholesterol	52.1	45–65 mg/dl
LDL-Cholesterol	170.3 ↑	< 130 mg/dl
Total Cholesterol/HDL ratio	4.5	< 5:1

eGFR: estimated glomerular filtration rate, T3: triiodothyronine, HDL: high-density lipoprotein, LDL—low-density lipoprotein

### Clinical course

**Figure 2:**
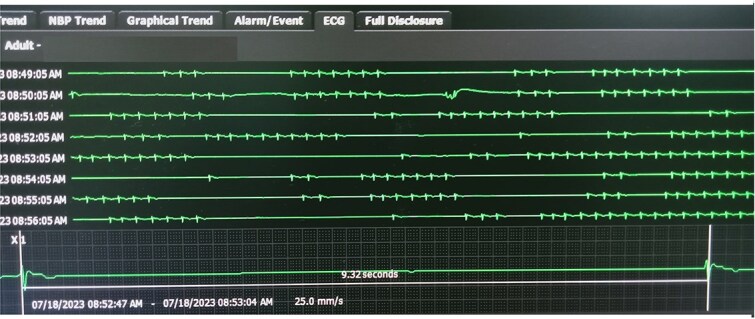
ECG monitor showing several episodes of sinus pause and sinus arrest.

**Figure 3:**
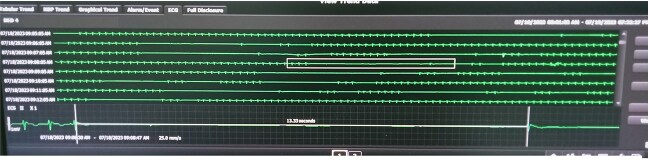
ECG showing maximum sinus arrest of 13.33 second.

She was admitted to the coronary care unit. During cardiac monitoring, she experienced several episodes of sinus pause and sinus arrest (Fig. 2). She experienced three episodes of transient loss of consciousness (each < 30 seconds), accompanied by generalized tonic clonic jerks, coinciding with sinus arrest (longest 13.33 sec). (Fig. 3). Since bradyarrhythmia associated with loss of consciousness and seizures typically point to cardiogenic syncope, neurological work-up such as CT brain or electroencephalogram were not done.

Urgent temporary external transvenous pacing was initiated via the femoral vein with a ventricular rate of 60/min due to symptomatic sinus arrest. With consideration of hypothyroid-induced bradycardia, possibly precipitated by Escitalopram, cold weather, and a recent flu-like infection, she was treated with Levothyroxine 100 μg daily. Escitalopram was discontinued after discussion with a psychiatrist.

**Figure 4:**
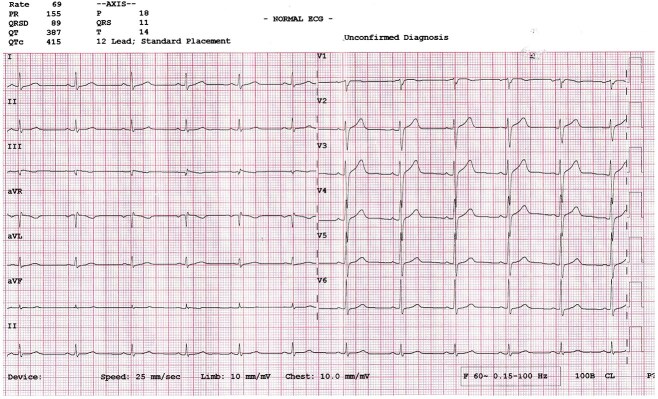
ECG showing sinus rhythm with heart rate 69/min.

After 10 days, her heart rate stabilized at approximately 65/min without pacing. Continuous ECG monitoring demonstrated normalization of sinus rhythm, with complete resolution of bradycardia and sinus arrest. The seizures did not recur after cardiac stabilization. Thus, temporary pacing was subsequently removed. She was discharged on the 12^th^ day of admission. Education was provided regarding the importance of regular Thyroxine compliance. Follow-up at four weeks showed a heart rate of 69/min without recurrence of bradycardia or sinus arrest (Fig. 4). At three months, she was clinically and biochemically euthyroid.

## Discussion

Thyroid hormone, triiodothyronine (T3), exerts its physiological effects by binding to intracellular thyroid receptors, regulating gene expression and protein translation. It increases basal metabolism and influences cardiac output and myocardial contractility via effects on sarcoplasmic reticulum calcium (Ca+) release and myosin heavy-chain adenosine triphosphate (ATP) activity [1]. Thyroid hormone also has a permissive and synergistic action on catecholamines by upregulating the β-adrenergic receptors on the cell membrane of cardiac myocytes [4]. Moreover, triiodothyronine is responsible for the cellular membrane depolarization-repolarization cascade by modulating the sodium and potassium permeability and increasing the L-type calcium channel in the sino-atrial node [5]. Additionally, localized edema caused by hypothyroidism may compress the atrioventricular (AV) node, impairing conduction [6].

Therefore, hypothyroidism leads to impaired cardiac inotropic and chronotropic functions and endothelial-mediated vasorelaxation. Hypothyroidism is associated with various ECG changes, including sinus bradycardia, prolonged QT interval, low-voltage QRS complexes, flat or inverted T waves, AV blocks, and sinus node dysfunction, including sinus arrest and sick sinus syndrome. In severe hypothyroidism, these features are more pronounced and can evolve into myxedema coma, characterized by hypothermia, hypoventilation, and multi-organ failure [2, 7].

Because of its vague characteristics, recognizing hypothyroidism early and initiating thyroxine replacement therapy is crucial. TSH remains the most reliable marker for adequacy of replacement therapy, with a target within 0.4 to 4.0 μIU/L [8]. Cardiac complications secondary to hypothyroidism are usually reversible with exogenous thyroid hormone replacement. In acute myxedema crisis, intravenous levothyroxine is preferred due to faster onset, whereas oral therapy takes weeks to show effects [9]. A temporary pacemaker may be required in symptomatic bradycardiac patients. In our case, there was no intravenous thyroxine available, and due to cardiovascular instability, temporary cardiac pacing was applied while awaiting the effect of oral levothyroxine.

In addition, the role of medication such as escitalopram, which has been associated with bradycardia, was considered. Several studies suggest that SSRIs like escitalopram can cause bradycardia in some patients [10]. On the other hand, the seasonal variation of serum TSH levels has been documented, with higher levels observed during colder seasons, possibly due to increased cold exposure stimulating hypothalamic–pituitary-thyroid axis activity [3]. In our region (Southeast Asia), the monsoon season (average temperature 22–26°C) may similarly exacerbate hypothyroid symptoms compared to warmer months (30–35°C). So, patients with hypothyroidism suffer more symptoms in winter or monsoon season. Our patient suffered from prolonged sinus arrest in monsoon season (local temperature is 25’C). On that account, it was assumed that our patient’s sinus arrest episodes were likely precipitated by a combination of longstanding hypothyroidism, cold weather, recent viral illness, and escitalopram use.

## Conclusion

Hypothyroidism is common, especially among the elderly. Early diagnosis and management are vital, as most cardiovascular complications of hypothyroidism are reversible with thyroid hormone replacement. The decision to implant a permanent pacemaker should be individualized, considering reversible factors like hypothyroidism. Comprehensive clinical assessment including precipitating factors for an acute hypothyroid crisis, and laboratory investigation of thyroid status should not be delayed to avoid unnecessary invasive procedures. Over and above that, patients should be given health education about the importance of regular Thyroxine compliance and precipitating factors of acute hypothyroid crisis.

### Take away messages

1. Hypothyroidism can lead to life-threatening bradyarrhythmias, including sinus arrest and sick sinus syndrome.
2. Clinicians should consider thyroid dysfunction in patients presenting with unexplained bradycardia or syncope.
3. Multiple factors (medication noncompliance, seasonal temperature changes especially cold weather, concurrent medications, and acute illness) can precipitate severe cardiovascular complication in hypothyroid patients.
4. Temporary pacing may be needed while awaiting thyroid hormone effects.

## Supplementary Material

CARE_checklist-Copy_omaf247

## Data Availability

Data available on request due to privacy/ethical restrictions.
